# Appearance-related stigma and its implications for genetic counselling practice

**DOI:** 10.1007/s12687-026-00905-8

**Published:** 2026-06-03

**Authors:** Kerry Montgomery, Diana Harcourt

**Affiliations:** Centre for Appearance Research, School of Social Sciences, College of Health, Science & Society, University of the West of England, Frenchay Campus, Bristol, BS16 1QY UK

**Keywords:** Visible difference, Stigma, Psychological, Genetic counselling, Training

## Abstract

In an appearance conscious society, individuals with genetic conditions that affect appearance often face unique psychosocial challenges that can shape reproductive decision-making. While research has highlighted the role of stigma in shaping decision-making, little is known about how genetic counsellors perceive and address appearance-related concerns in clinical practice. This study explored the experiences of UK-based genetic counsellors working with individuals affected by congenital conditions that alter appearance, with a focus on reproductive decision-making. Using a qualitative design, six genetic counsellors across different genetic specialties were recruited and interviewed. Data were analysed using reflexive thematic analysis revealing three key themes. The first theme highlighted genetic counsellors’ perceptions of how social and emotional factors (particularly guilt, shame and lived experience) influenced reproductive choices, with patients often expressing concern about passing on conditions associated with stigma. The second theme explored a perceived reluctance among patients to discuss appearance-related concerns which in turn made counsellors cautious about raising such topics. The third theme identified a need for more inclusive and accessible information and support, alongside enhanced training for counsellors in addressing appearance-related stigma. Participants emphasised the value of lived experience in shaping practice and called for greater awareness of the genetic counselling role to improve accessibility. These findings inform future training and support within genetic counselling.

## Introduction

In an appearance-conscious world, it is well established that conditions or injuries which affect appearance (often referred to as visible differences) can have a significant impact on psychological well-being and quality of life (Fournier et al. 2023; Feragen and Stock 2017; Rumsey and Harcourt 2004). Conditions that affect appearance may be congenital (inherited from an affected parent or arising from a *de novo* genetic alteration) or acquired (for example through injury, illness, medical or surgical intervention). Research suggests the psychological impact of visible difference is not determined by whether the condition is congenital or acquired. Optimism, social support and disguisability of the difference have been identified as significant predictors of psychosocial outcomes in adulthood (Zucchelli et al. 2023).

A challenge that is unique to congenital visible differences (e.g., Crouzon Syndrome, Achondroplasia) is the possible inheritability of the condition. Parents report concerns about potential children having the same condition (Ardouin et al. 2021), and some evidence suggests that women with cleft lip and palate are less likely to have children than those without a cleft (Yttri et al. 2011). Parents of children with inheritable conditions that affect appearance often report feelings of anxiety and guilt (Clarke 2016; O’Hanlon et al. 2012), experiences that are also found across other genetic conditions. These emotions may be intensified where conditions are not life-limiting, as decisions about having children are often more uncertain and morally complex (Frigon et al. 2022).

Whilst genetic conditions affecting appearance can vary widely in their medical implications, the visible aspect should not be underestimated and is often one of the most significant challenges impacting quality of life (Crawford et al. 2015; Fournier et al. 2023; Stock and Rumsey 2015). Negative reactions from others, including stigma and discrimination, are commonly reported in accounts of individuals’ experiences of living with a visible difference (Fournier et al. 2023; Myhre et al. 2021; Rustad et al. 2025).

Stigmatisation can be understood as a social and cultural construct in which individuals are marked as different due to their perceived deviation from societal norms (Goffman 1997), resulting in the individual being devalued and experiencing a loss of social status (Pescosolido and Martin 2015). Goffman’s perspective aligns closely with the social model of disability (Oliver 2013), which posits that individuals are not disabled by their condition but by societal barriers such as discrimination. These social challenges are therefore highly relevant for genetic counsellors to consider (Oliveira et al. 2025).

The Social Stigma Framework (Pryor and Reeder 2011) outlines four different forms of stigma, with Rasset et al. (2022) providing an overview of how these relate specifically to visible difference. Public stigma refers to cognitions and behaviours towards those who are stigmatised and highlights how the social environment can create significant challenges when someone looks different. Research consistently shows that people often hold less favourable opinions of individuals with visible differences (Stone and Wright 2012) and evaluate their personality attributes more negatively (Bogart et al. 2019). Self-stigma refers to the internalisation of these negative societal beliefs. This is particularly relevant in the context of reproductive decision-making, as decisions are shaped primarily by individuals’ personal experiences of their condition (Clarke 2016; Stock and Rumsey 2015). Rather than being based on objective assessments of severity, decision-making is more strongly influenced by how individuals feel about their condition and their psychological adjustment (Stock and Rumsey 2015), highlighting the interaction between public stigma and self-stigma. Stigma by association refers to the experience of stigma among those who do not have the condition themselves but are associated with someone who does, making this especially relevant for family members of individuals with a genetic condition. Structural stigma refers to the ways in which societal systems and representations perpetuate stigmatisation, with the media identified as a key source (Rasset et al. 2022). Understanding how these different forms of stigma operate in the context of visible difference is critical for genetic counselling practice, as it can help counsellors recognise how societal attitudes, internalised beliefs, and concerns about family members shape reproductive decision-making.

Individuals with inheritable conditions affecting appearance often report experiences of stigma across family, social, educational, workplace, and healthcare settings (Oliveira et al. 2025). Some individuals also describe how healthcare professionals may underestimate the psychological impact of altered appearance and overlook concerns related to family planning (Oliveira et al. 2025). Much of the existing literature concerning conditions that affect appearance has focused on psychosocial adjustment following diagnosis rather than on how concerns about appearance could influence reproductive decision-making. In particular, there has been limited exploration of how lived experience, anticipated stigma, and concerns about social judgement shape decisions about having children and the use of reproductive technologies. This represents an important gap, as reproductive decisions are not made solely on the basis of clinical information or condition severity, they are but are considered within broader social contexts. The current study aims to explore how genetic counsellors understand the social context in which decisions are made, and their perceptions of how individuals consider appearance within decision-making to offer clinically relevant insights into how social experience, identity, and stigma shape reproductive decision-making, with direct implications for genetic counselling practice, training, and the provision of holistic, psychosocial care.

## Method

### Design

The study was designed in collaboration with a Research Advisory Group comprising of individuals (*n* = 6) with an inheritable visible difference. A further advisory group was formed of experts by profession (*n* = 4) which included genetic counsellors, clinical geneticists, and neurofibromatosis specialist nurses. The Research Advisory Groups also included representatives from charities that represent a range of conditions that affect appearance. These groups played a central role in shaping the study design and choice of a qualitative approach. The semi-structured interview schedule was collaboratively developed with them to ensure that the questions were relevant and could meet the aims of the study. Ethical approval for the study was granted by a Research Ethics Committee at the University of the West of England.

### Recruitment

Participants were recruited between May and September 2024 via social media and professional networks including the Association of Genetic Counsellors and charities working with individuals with inheritable visible differences (see acknowledgements). The study advert contained details of the research, a short online demographic questionnaire and the researcher’s contact information. The questionnaire collected information related to the participant’s professional role including location, speciality and length of time working in genetics. Those who chose to take part engaged in a one-on-one, semi-structured interview conducted online through Microsoft Teams. Each interview lasted approximately sixty minutes. To thank them for their time, participants were offered the choice of a £25 online shopping voucher or a donation to a charity of their choice.

### Participants

Participants were eligible if they self-reported having experience of working with patients with a genetic condition affecting their appearance, in NHS services in the UK. No specific time frames or minimum level of experience were stipulated.

All participants (*n* = 6) identified as female and were employed as genetic counsellors or geneticists with experience levels ranging from pre-registration to department lead. Participants worked across several specialties including cancer, neurological conditions, and general genetics. They worked with individuals and couples considering genetic testing, exploring reproductive options, who were currently pregnant, or planning future pregnancies. Specific demographic details are not included in this paper in order to maintain participant confidentiality.

### Analysis

Interviews were recorded and transcribed using Microsoft Teams’ transcription feature, with the researcher reviewing and editing the transcripts for accuracy. All transcripts were pseudonymised and any identifiable data was removed. An inductive, qualitative approach was adopted to gain an in-depth understanding of a complex and largely understudied area, and interviews were analysed using Reflexive Thematic Analysis (RTA; Braun et al. 2022).

RTA was chosen as it emphasises the active role of the researcher in identifying patterns and meaning within the data. RTA is situated within a non-positivist framework that positions the researcher as a resource in the meaning-making process, rather than a threat to objectivity. It acknowledges that meaning is context-dependent and shaped by the researcher’s own perspectives and experiences. The first author has a genetic condition that affects appearance and accessed genetic testing 30 years ago. It is important to acknowledge the position of the first author as a ‘me-researcher’ (Bogart 2024) and the potential influence this may have had on participant responses. However, Bogart (2024) acknowledges the strengths of self-relevant research, highlighting that researchers with lived experience often have insight into gaps in the research that may not otherwise be explored. While the researcher’s role in meaning-making is central to RTA, care was taken to ensure that the analysis did not become a reflection of the researcher’s own narrative. A reflexive diary was maintained and completed after each interview and interviews were discussed in supervision with the second author. These discussions provided a space to explore the researcher’s emotional responses and to ensure that participants’ meanings were interpreted through, rather than overtaken by the researcher’s lens.

Braun at al (2022) six-step procedure for RTA was followed. Familiarisation with the data occurred naturally, as the researcher was responsible for transcription accuracy and anonymising the transcripts. This was followed by coding the transcripts using NVivo (version 14). Transcripts were coded individually and emerging themes from each transcript were carried forward and refined throughout the analysis of subsequent transcripts. This iterative process continued until a final set of themes captured patterns across the dataset. The final themes were then reviewed, with some being merged or restructured. Overarching themes and subthemes were used to organise the analysis, as this approach allowed for clearer storytelling and helped to keep related concepts together.

## Results

The current study focused on genetic counsellors’ perceptions of what influences reproductive decision-making for individuals with a condition that affects appearance which can be inherited. Participants shared specific experiences of supporting individuals with conditions affecting appearance whilst also talking about similarities and differences between appearance altering conditions and other genetic conditions. Throughout the interviews, genetic counsellors referred to the individuals they support as ‘patients’; therefore, we have adopted this terminology for consistency and pseudonyms have been used to refer to maintain anonymity of the genetic counsellors.

Three themes captured participants’ perceptions and experiences of providing genetic counselling for these individuals. Theme one explores the social and emotional factors that influence decision-making for individuals with conditions affecting appearance, drawing on genetic counsellors’ perceptions of how these decisions are shaped by wider societal pressures rather than made in isolation. Theme two explored genetic counsellors’ perceptions of stigma related to visible difference and the ways in which this stigma can shape the genetic counselling process, and theme three explored the need for training to support genetic counsellors in addressing appearance-related stigma.

### Theme one: Social and emotional factors influencing decision-making by individuals with conditions affecting appearance

Genetic counsellors reflected on their perceptions of how a range of social and emotional factors, such as feelings about looking different, patients’ social experiences, feelings of guilt, and the differing implications of child- versus adult-onset conditions, shaped the conversations they had with patients. This theme explores how these factors influenced the decision-making process from the perspective of genetic counsellors.

Participants discussed how guilt was a central topic in many genetic consultations. Considering conditions which affect appearance, guilt often stemmed from parents not wanting their child to go through the psychosocial challenges they themselves had experienced. Participants also described a sense of guilt amongst patients considering options like pregnancy termination or Pre Implantation Genetic Testing (PGT). Genetic counsellors described decision-making as a double-edged sword, where parents and/or prospective parents would experience guilt no matter what choice they made. Helping patients manage this guilt was seen as one of the key aspects of the genetic counsellors’ role.

“*It’s sort of one of the first things I say is none of this is your fault. You didn’t do anything to cause this and it’s made people start crying before*,* that sort of relief of hearing it from someone else*,* of being absolved of the guilt” Harriet*.

Participants acknowledged the challenge of making decisions related to appearance, as these choices may be perceived as superficial, and noted a perception of greater guilt when patients are considering appearance in reproductive decision-making compared with medical factors.

*“I suspect that there is greater challenge and greater guilt when it is something that could be viewed as cosmetic or superficial - something to do with appearance rather than medical suffering” Maria*.

Maria also highlights the distinction between the unavoidable medical realities of a condition like Huntington’s disease and the perception of preventable social suffering experienced by those with a visible difference arising because of stigma and discrimination.

*“The medical stuff of Huntington’s is unavoidable until we get a cure. The suffering of visual difference shouldn’t be unavoidable*,* society does not have to do that to people and it’s really upsetting that it does” Maria*.

Reproductive decision-making was influenced by age of onset of the condition in question. Genetic counsellors distinguished between adult-onset conditions (e.g., Huntington’s disease) and those that manifest in infancy or early childhood (e.g., cleft lip and palate). Conditions that affect appearance are often visible from birth, prompting parents to focus their concerns on their child’s early development and wellbeing. In contrast, adult-onset conditions typically do not present until later in life which for some parents might allow for a sense of emotional detachment and the possibility for the child to make autonomous decisions as an adult.

*“I do quite a lot of cancer work and the majority of those*,* there aren’t significant risks in childhood so when we’re talking about having children*,* we’re talking about the implications for them as adults when they have to start facing that risk… and as an adult they could make a decision about whether they want to know and whether they would want testing*,* screening or risk reducing treatments and parents often take comfort in that thinking*,* I don’t have to worry about my children as children” Lucy*.

Decisions are often shaped by our life experiences, and reproductive choices are no different. Participants discussed how social interactions and the responses of others had a significant influence on reproductive decision-making:

*“I think it is entirely their lived experience*,* if they’ve had an OK time with it [the visible difference]*,* then they’re like*,* yeah*,* not a problem but if they’ve had whatever going on*,* they’ve struggled with accessing the healthcare appointments or with how others interact or see them or how much support they’ve managed to get*,* definitely that does change their views on pregnancy” Harriet*.

When discussing the reasons why patients had opted for genetic counselling and were considering their reproductive options, genetic counsellors in the current study described patients wanting to avoid their child experiencing the same social challenges or bullying that they had experienced:

*“He had adapted things for his life*,* the car and things but talked about how much harder it [appearance] made work and how much harder it made going to school and being around boys and bullying and just went “I don’t want to put a child through that”” Harriet*.

Genetic counsellors felt that patients’ experiences of appearance-related bullying had a significant influence on their reproductive decision-making. In some cases, as in this quote from Maria, genetic counsellors reported how social challenges were the key factor when considering reproductive options.

*“My impression for this person certainly was that going through some surgeries when young was fine*,* family support was good*,* he was someone who hadn’t had any real major kind of health problems because of it. It wasn’t affecting his breathing*,* his sort of his hand signs were really*,* really mild. It was mainly just facial difference*,* and he was sort of quite OK but when we dug a little deeper*,* he said “I did get bullied really badly*,* that’s why I went and had surgeries”” Maria*.

In the quote below, a genetic counsellor reflects that acceptance of a condition is shaped by the patient’s life experiences, and other people’s reactions to their visible difference may influence how they feel about the possibility of having a child with the same condition.

*“I think it’s not particularly what they mentioned*,* but I get the impression that how accepting they are of their own condition or diagnosis can be very personal and that equally that’s based on experience throughout their lives and how people have responded to their physical differences as well as how they would then perceive the potential of having a child with a similar condition” Sarah*.

Maria also reflected on the stigma surrounding parenthood for individuals with a genetic condition. While she did not explicitly state that having children may be frowned upon, her comments seemed to suggest an underlying perception that such decisions are often met with stigma.

*“I think sometimes people feel that they need to justify that they feel guilty about just wanting to have a baby the way everyone else does (natural pregnancy)*,* but also a societal stigma if you have a condition and you reproduce because*,* well*,* you can’t win” Maria*.

In summary, genetic counsellors perceived that considering appearance within reproductive decision-making could feel especially challenging for patients, sometimes carrying an added layer of guilt because such choices may be viewed as more “superficial”. They also recognised that difficult social experiences, such as stigma or bullying, shaped patients’ fears about how a future child might be treated, influencing how they approached their reproductive options.

### Theme two: Stigma, visible difference and the role of the genetic counsellor

Theme two explores genetic counsellors’ perceptions of stigma associated with visible difference and how this influences the genetic counselling process. Maria described how, in cases where the primary challenges associated with a condition relate to appearance and difficult social experiences, these issues can be particularly hard to address in counselling. She reflected on the discomfort around acknowledging the impact of appearance, noting that *“no one wants to talk about the fact that how you look can affect the way life treats you and you might make decisions about that.”*

Differences between conditions affecting appearance and other types of genetic conditions were discussed. As one participant noted, *“I think there is something very different about visible differences to other genetic conditions… for a child who’s struggling with it they look in the mirror and they have the evidence that they look and feel different.” Anna*.

One genetic counsellor talked about differences of sex development (DSD), highlighting the role of societal stigma as a driving force behind the difficulties experienced. As they explained, *“I think there are things where I feel that the driving force is societal stigma… there’s so much stigma around anything to do with sex and gender and being normal and I think the same with things which are primarily about appearance.” Maria*.

In the quote below, another counsellor expressed that society is not designed to accommodate people who look different, and this lack of inclusivity shapes how individuals experience daily life. This highlights the importance for genetic counsellors to recognise and understand how stigma and social exclusion can be an integral part of a patient’s lived experience.

*“…it’s not just the painful idea of bullying as a child… actually sometimes the world is just not set up for people with other differences*,* and so it’s not very accepting of people who look different or who move differently… you’re always aware of it and it always informs your daily life.” Anna*.

Genetic counsellors described a hesitancy from patients to discuss appearance-related concerns during appointments. One participant, Sarah, suggested this reluctance may stem from feelings of shame.

*“Some people say it a bit more tentatively*,* I do wonder if some people don’t say it because of that worry of the shame*,* and there’s probably times that maybe I should have probed more about how patients would feel about it” Sarah*.

Participants in this study described a tension between acknowledging the role of stigma and appearance in reproductive decision-making, and the patient’s potential reluctance to discuss these issues. This reluctance can make counsellors hesitant to raise the topic, fearing they might inadvertently reinforce the stigma surrounding it. However, not acknowledging the impact of stigma, and thus its influence on decision-making, could have a negative impact on the genetic counselling process.

*“I’m led by how much patients want to talk about it and maybe there’s an aspect of if a patient’s experiencing that themselves they’re not concerned*,* outwardly*,* explicitly*,* is it insensitive for me to then be asking that?” Lucy*.

Jen identified a potential training need for genetic counsellors, suggesting that hearing directly from individuals with lived experience could enhance understanding of how to sensitively explore appearance-related concerns and how these might influence reproductive decision-making. She reflected on the challenge of knowing when and how to raise these topics, especially when patients may feel shame or guilt. Jen questioned whether counsellors should gently probe further, even when patients seem hesitant, in order to better support them, emphasising that guidance from those with lived experience would be invaluable in navigating this discomfort.

*“I’m led by patients*,* but if you talk to people (with lived experience) and they feel like they’re not saying things because of worry of shame or guilt should I (ask questions)? Is the patient’s perspective that they’d like to be probed a little bit more about these things? Do I have to just push past that comfort to get the main that sort of discomfort in me about asking these things to get the best for my patient? I would love to hear that” Jen*.

Part of the role of genetic counsellors is to discuss prenatal testing options available to families. One counsellor described how a couple had wanted to find out if their child was affected ahead of the birth so they could focus on bonding with their child.

*“Sometimes couples are very like I’m very happy to have a child affected in the same way as I am but that pressure of that moment and something about taking away from that moment of first holding baby. I think there’s also a little bit of a sense of guilt there and just how they might feel in that moment ahead of time” Jen*.

Genetic counsellors recognised the psychological need many parents have to feel prepared, not only to avoid the shock of an unexpected diagnosis but also to preserve the joy of birth, rather than having it overshadowed by the discovery of their baby’s condition. This need may be especially pronounced in conditions that affect appearance, as these are often visible at birth and therefore must be acknowledged and addressed by the midwifery team.

*“I also did the Cleft Lip and Palate clinic and I would often talk to those patients about family planning and you can’t always tell cleft lip and palate from a scan. Sometimes you can tell but what patients would often do is go for a 4D scan*,* which can sometimes show the palate as well*,* again*,* just for that information so that they can prepare because parents would often feedback that it was just such a shock for their first child*,* if they had it as well*,* that they just they want to know ahead of time” Anna*.

In conclusion, having a genetic condition that affects appearance can raise challenges that differ from those associated with non-visible conditions. Focusing on appearance may feel uncomfortable or be perceived as superficial, and genetic counsellors described sometimes finding it difficult to raise these issues in sessions, despite recognising their importance in supporting families. Social challenges, including stigma and past negative experiences, were also seen to influence reproductive decision-making, with patients often expressing a desire to protect a future child from experiencing similar social difficulties. Genetic counsellors described their role as supporting families to navigate emotionally complex decisions by balancing patient-led practice with the challenge of knowing when to gently explore appearance-related issues that patients may hesitate to raise due to feelings of shame or guilt. In discussions about prenatal testing, counsellors positioned themselves as helping parents prepare emotionally for birth and early bonding, particularly when conditions are visible, so that reproductive choices support both informed decision-making and psychological preparedness.

### Theme three: Designing inclusive tools for genetic counselling and patient support

Genetic counsellors in this study identified several areas where additional training and support could help them better support individuals and families affected by conditions that alter appearance. Participants described challenges in raising appearance-related concerns, exploring difficult social experiences, and addressing worries about a future child, suggesting that further guidance on how to initiate and navigate these conversations would be beneficial. Genetic counsellors also highlighted gaps in available resources, particularly in relation to clear information about genetic counselling and how families can access support. Anna reflected on this need, stating:

*“I think my ideal situation is that everybody with a genetic condition knows how and where to access genetics care and that might be on their charity websites. It might be elsewhere*,* somewhere almost at the point of diagnosis” Anna*.

Genetic counsellors described having had varied exposure to conditions affecting appearance during their training and in their clinical role.

*“We definitely learn about specific conditions*,* but there are just endless genetic conditions*,* and we obviously can’t learn about all of them… they focused on the most common or the most classic genetic conditions” Anna*.

They also reflected on how appearance remains a taboo and often uncomfortable topic to address in clinical conversations. As highlighted in the quote below, participants felt that professionals are typically more at ease discussing the medical aspects of genetic conditions. This suggests a need for greater support and training to help counsellors navigate the sensitive, yet important, conversations around appearance.

*“I feel like it’s a bit of a taboo. I still don’t think we talk enough about this (appearance) because like many stigmatised or uncomfortable topics*,* people are worried about getting it wrong and they’re not sure how to approach it and they get very comfortable talking about medical things*,* because that’s OK you’re allowed to make choices*,* but no one wants to talk about the fact that actually*,* how you look can affect the way life treats you and you might make decisions about that” Maria*.

The most relevant learning genetic counsellors had received was hearing directly from individuals with genetic conditions that affect appearance.

*“The most valuable information we can ever have is information from those particular communities… their voices are always the most important part.” Jen*.

The importance of the patient voice was also noted in service design and delivery with one participant highlighting the need for feedback to help improve genetics services and what can be offered.

*“We don’t see the whole picture. Hearing what people have found useful or not useful helps us to change our service and what we’re able to offer.” Sarah*.

Although the study focused on conditions affecting appearance, genetic counsellors in this study reflected more broadly on the accessibility of genetic information. They emphasised a general need for clear, user-friendly resources across all conditions.

*“A lot of what’s out there is jargon. It’s research paper words and research paper language which isn’t easily readable.” Harriet*.

One genetic counsellor reflected on an experience where they provided information about support organisations to a patient with a visible difference. This prompted them to question whether it was the right thing to do, or whether they had unintentionally drawn attention to an issue the patient had not considered problematic.

*“I remember looking up and checking my resources for support organisations for visible difference and I can’t remember what resources I actually gave them because I think it’s always such a delicate one of if somebody is happy where they are*,* they’ve reached a decision*,* and this is what they’re saying is right for them. There’s a real risk of invalidating that by giving them resources that run counter of saying*,* well*,* no*,* but you shouldn’t feel that way and especially around prenatal testing” Maria*.

Genetic counsellors also talked about wanting the general public to know more about their role and what it involves, suggesting there is still work to do on raising awareness of genetic counselling more widely.

*“I’d love people to know what genetic counsellors do and that they feel approachable because often you don’t know what a genetic counsellor is until you get an appointment booked with one of them” Anna*.

There was also a suggestion that people would benefit from learning more about genetics earlier and promoting an open dialogue about the condition within families.

*“I think probably resources from my point of view would be things for families to sow those seeds earlier on about saying how to keep talking to your child about these things as they grow up*,* you know*,* not we had your surgery and it’s fine we can all put it in a box and pretend it didn’t happen. But how do you keep normalising and supporting that so that they have the opportunity to work through it when they’re a bit older and think about what it means.” Maria*.

This theme highlights genetic counsellors’ perceptions of the training and resource needs required to support individuals and families affected by a condition that affects appearance. Participants reflected on how appearance-related stigma and anticipated judgement could shape patients’ willingness to raise social concerns, while also contributing to counsellors’ own uncertainty about when and how to initiate these discussions. Suggestions for improving practice focused on practical strategies, including clearer signposting to relevant organisations, increasing awareness of genetic counselling within visible difference communities, and opportunities for individuals to hear from others in similar situations. While counsellors noted that some of these needs were relevant across genetic conditions more broadly, they were seen as particularly salient where conditions are visible.

## Discussion

This study explored the experiences of genetic counsellors supporting individuals with genetic conditions that affect appearance. Although looking different can have a significant psychosocial impact, there is limited research that specifically examines how these social challenges influence the practice of genetic counselling.

Theme one explored genetic counsellors’ perceptions of how social and emotional factors, particularly feelings of guilt, shame, and lived experience influence reproductive decision-making. Genetic counsellors reported that patients often expressed guilt related to the possibility of passing on their condition, largely driven by concerns that their child might face the same social stigma, bullying, and psychosocial challenges that they had experienced. These emotional responses were seen as powerful motivators in shaping reproductive choices including whether patients chose to pursue IVF-PGT. This finding is in line with previous research emphasising how social experiences shape attitudes toward having a child with a visible difference (Clarke & Wallgren-Pettersson 2019; Stock and Rumsey 2015).

Feelings of guilt and fears of transmission are not unique to conditions that affect appearance. For example, a recent systematic review found that reproductive decision-making in the context of Huntington’s disease (HD) is often shaped by these same concerns (Fahy et al. 2023). However, the key distinction lies in the underlying nature of these fears. In HD, the concern is primarily about the possibility of passing on a life-limiting genetic condition. In contrast, for conditions that affect appearance, while medical considerations remain important, prospective parents are often equally, if not more, concerned about the social challenges and stigma their child may face (Stock and Rumsey 2015). Considering these findings in line with the Social Stigma Framework, these concerns reflect the impact of anticipated public and structural stigma, alongside the internalisation of these beliefs through self-stigma. Reproductive decision-making is shaped not only by genetic risk but by expectations about social responses to visible difference and the desire to protect future children from negative social experiences. For genetic counsellors, recognising the role of stigma is crucial, as it enables a more in-depth understanding of patients’ motivations and supports empathetic counselling that addresses emotional, social, and psychological influences alongside medical information.

In theme two genetic counsellors noted a perceived reluctance from some patients to discuss appearance concerns which could make counsellors themselves hesitant or cautious about broaching appearance-related topics. This reluctance can be understood through the social stigma framework (Pryor and Reeder 2011) whereby genetic counsellors might anticipate public stigma surrounding visible difference and seek to avoid causing offence which can inadvertently mean that appearance becomes a taboo topic. There is a need for genetic counsellors to be aware of the potential impact of stigma to ensure that their practice does not inadvertently reinforce it (Gaff and Clarke 2007). Acknowledging the tension between respecting patient boundaries when certain topics are not raised, while also asking questions in a way that creates safety and trust, will enable patients to explore sensitive issues.

Prenatal diagnosis can help families prepare emotionally and practically, reducing the potential shock of a child being diagnosed at birth. Research highlights the importance of the immediate postnatal period for fostering secure attachment, with skin-to-skin contact playing a key role (World Health Organization 2020). If a diagnosis is unexpected or distressing, for example if they weren’t aware the baby would have a visible difference, it may disrupt this bonding process, emphasising the need for sensitive support in the delivery room.

The final theme explored the need for more inclusive, sensitive, and accessible information and support. Genetic counsellors described having little confidence in addressing appearance concerns, noting that these topics often felt harder to raise than medical issues. This may reflect structural stigma within healthcare systems, where medical concerns are more readily discussed, while appearance-related concerns and associated psychosocial difficulties receive less attention, with consequences for the quality of patient support.

Despite acknowledging how busy genetics departments are, counsellors felt there was a need for people to understand their role in order to make genetic counselling more accessible. They also identified a need for training on the psychosocial impact of conditions affecting appearance in the context of genetic counselling, which would benefit from incorporating first-hand perspectives of individuals with lived experience. Such perspectives were viewed as the most valuable learning resource across genetic conditions, highlighting the importance of integrating patient voices.

The findings of this study emphasise that stigma related to visible difference significantly shapes reproductive decision-making and counselling interactions. While it may feel uncomfortable to acknowledge, patients often factor in anticipated societal responses when considering having a child with a visible difference. It is therefore crucial that genetic counsellors recognise the role society plays in shaping decisions.

This study has several limitations. Firstly, the number of participants was small; however, recruitment in rare conditions is often challenging. While it is important to acknowledge the limited sample size, it is equally important not to overlook that qualitative research prioritises the depth and richness of the data. Future research could focus on specific conditions, allowing smaller samples to represent a more homogeneous population. In addition, participants were at varying stages of their careers, with some having worked extensively in specialist clinics for visible conditions such as cleft lip and palate or Neurofibromatosis, while others had more limited exposure. Although all participants met the inclusion criteria by having experience in these clinics, for some this was not a prolonged period, as much of their career had been spent in areas such as cancer genetics or neurodevelopmental disorders. Future research could benefit from engaging a broader and more diverse group of genetic counsellors, including international settings, to explore potential similarities and differences in support practices across healthcare systems and cultural contexts.

## Conclusion

While appearance may be widely recognised as an important factor for patients, there has been a lack of research into how it influences reproductive decision-making by people with inheritable visible differences, and how genetic counsellors currently manage such discussions with their patients. The findings of this study, which interviewed 6 professionals with experience of providing genetic counselling and support, suggest that stigma related to looking different play a significant role in shaping patients’ reproductive decisions. The influence of appearance on reproductive choices needs to be considered by genetic counsellors, in order to understand the broader social context in which these decisions are made. While it may feel uncomfortable to acknowledge that such decisions are informed by how society may respond to a child with a visible difference, this reality must be recognised. Understanding these dynamics is critical to ensuring that reproductive counselling is empathetic, informed, and responsive to the social contexts patients navigate.

The study findings underscore the vital role of genetic counsellors in recognising and navigating the social stigma surrounding visible difference and its influence when making reproductive decisions. By creating a safe, non-judgemental space for these conversations and ensuring their approach addresses both medical and psychosocial needs, professionals p can support patients who have inheritable genetic conditions that impact on appearance in making informed reproductive decisions.

## Data Availability

The data supporting the findings of this study are qualitative and contain potentially identifiable information. To protect participant confidentiality, the full dataset is not publicly available. However, de-identified excerpts may be available from the corresponding author upon reasonable request and subject to appropriate ethical considerations **.**.
